# Effectiveness of Hydrated Sodium Calcium Aluminosilicates and Discarded Date Pits as Dietary Adsorbents for Aflatoxin B1 in Enhancing Broiler Chicken Productive Performance, Hepatic Function, and Intestinal Health

**DOI:** 10.3390/ani14142124

**Published:** 2024-07-21

**Authors:** Ala E. Abudabos, Riyadh S. Aljumaah, Abdulaziz A. Alabdullatif, Ali R. Al Sulaiman, Zafar Hakmi, Abdulrahman S. Alharthi

**Affiliations:** 1 Department of Animal Production, College of Food and Agriculture Sciences, King Saud University, P.O. Box 2460, Riyadh 11451, Saudi Arabiaarsuliman@kacst.gov.sa (A.R.A.S.); zaferhakami@gmail.com (Z.H.); 2 Department of Agriculture, School of Agriculture and Applied Sciences, Alcorn State University, 1000 ASU Drive, Lorman, MI 39096-7500, USA; 3 Environmental Protection Technologies Institute, Sustainability and Environment Sector, King Abdulaziz City for Science and Technology, P.O. Box 6086, Riyadh 11442, Saudi Arabia

**Keywords:** aflatoxin adsorbents, aflatoxin B1, broiler, intestinal health, liver function, performance

## Abstract

**Simple Summary:**

Aflatoxin B1 (AFB1) is a potent mycotoxin that can severely harm broiler health and performance, leading to reduced growth rates, compromised liver function, and weakened immune function. The study explored the effectiveness of two dietary supplements, hydrated sodium calcium aluminosilicates (HSCASs), and discarded date pits (DDPs) in reducing the harmful effects of AFB1 on broiler chickens. The research involved feeding 240 chickens with different diets: a control diet, a control diet with AFB1, and an AFB1-contaminated diet with either HSCAS or DDP. The results showed that including HSCAS or DDP in the diet improved the chickens’ body weight, feed efficiency, and overall health. These improvements were evident through better liver and kidney function, higher protein and glucose levels in the blood, and enhanced digestive efficiency. Remarkably, DDP supplementation also reduced liver size and improved total antioxidant capacity levels, while HSCAS increased protein digestibility. The study concluded that both HSCAS and DDP are effective in reducing the negative impacts of AFB1 on broiler chickens, suggesting they could be valuable natural feed additives in poultry farming to ensure healthier birds and safer food products for consumers.

**Abstract:**

The research aimed to evaluate how effective hydrated sodium calcium aluminosilicates (HSCASs) and discarded date pits (DDPs) are as dietary adsorbents for aflatoxin B1 (AFB1) in enhancing the performance and health of broiler chickens aged 16 to 30 days. A total of 240 Ross 308 straight-run broilers were randomly allocated into four dietary groups, each with 10 replicates: a control diet, a control diet with 1000 ppb AFB1, an AFB1-contaminated diet with 0.5% HSCAS, and an AFB1-contaminated diet with 4% DDP. Incorporating HSCASs or DDPs into the AFB1-contaminated diet resulted in significant improvements across various parameters, involving increased body weight, improved feed conversion ratio, higher dressing percentage, decreased relative weights of kidney and spleen, elevated serum levels of total protein, globulin, and glucose, reduced serum alanine aminotransferase activity, and heightened hepatic protein concentration and glutathione peroxidase activity, along with diminished hepatic malondialdehyde content and glutamic oxaloacetic transaminase activity. Moreover, both supplements led to increased ileal villus height and surface area, enhanced apparent nitrogen-corrected metabolizable energy digestibility, and decreased AFB1 residues in the liver and kidney. Moreover, the dietary inclusion of DDPs significantly decreased relative liver weight, raised serum albumin concentration, lowered serum alkaline phosphatase activity, enhanced hepatic total antioxidant capacity level, and augmented ileal villus width. Conversely, the dietary addition of HSCASs significantly heightened apparent crude protein digestibility. In conclusion, the inclusion of HSCASs and DDPs in AFB1-contaminated diets can mitigate the toxic effects of AFB1 on broiler chickens, with DDPs exhibiting additional advantages in optimizing liver function and gut morphology.

## 1. Introduction

Aflatoxin B1 (AFB1), a toxic metabolite produced by *Aspergillus flavus* and *Aspergillus parasiticus*, is one of the most potent naturally occurring carcinogens [1]. It poses significant risks to animal health and productivity, particularly in poultry, where the contamination of feed is a prevalent issue [2]. Broiler chickens are highly susceptible to the adverse effects of AFB1 due to their rapid growth rates and high feed intake [3]. Recent studies have highlighted the multifaceted impacts of AFB1 on broiler chickens. AFB1 contamination has been shown to detrimentally affect growth performance by reducing feed intake and body weight while worsening the feed conversion ratio [4]. Carcass characteristics are also negatively influenced, with reductions in breast muscle yield and increased fat deposition [5]. Blood biochemical indices, such as serum protein levels and enzyme activities, are often altered, reflecting systemic toxicity and impaired metabolic functions [6]. Furthermore, AFB1 exposure compromises liver antioxidant status, leading to oxidative stress and damage to liver tissues [7]. This oxidative damage is mirrored in elevated levels of liver function enzymes, indicating hepatotoxicity [8]. The intestinal morphology of broilers is also adversely affected, with changes in villus height and crypt depth, which impair overall gut health [9]. Nutrient digestion and absorption are consequently reduced, exacerbating the negative effects on growth and performance [10]. The accumulation of aflatoxin residues in different tissues of the birds raises additional concerns regarding food safety and the potential for human exposure through the consumption of contaminated poultry products [11]. Therefore, effective strategies to mitigate AFB1 toxicity in broiler diets are of paramount importance.

One promising strategy to counteract the effects of AFB1 is the use of aflatoxin adsorbents in poultry diets [12]. Among these adsorbents, hydrated sodium calcium aluminosilicates (HSCASs) stand out as a group of naturally occurring minerals that include various forms of zeolites, bentonites, and other aluminosilicate clays [13]. These minerals have a crystalline structure composed of interconnected alumina and silica tetrahedra, creating a network of channels and cavities [14]. The presence of exchangeable cations such as sodium and calcium within this structure bolsters the adsorptive properties of HSCASs. The large surface area and high cation exchange capacity of HSCASs enable them to bind aflatoxins with high affinity within the pores and interlayers of their structure [15]. As a result, the bioavailability of aflatoxins in the gastrointestinal tract is considerably lowered, minimizing their absorption into the bloodstream and subsequent distribution to target organs [16]. A study by Yiannikouris et al. [17] demonstrated that the use of HSCASs across various in vitro and ex vivo models significantly reduced the bioavailability of AFB1 by over 60%, forming a protective barrier on the intestinal mucosa and limiting the toxin’s transmembrane transfer. Another study by Chen et al. [18] revealed that incorporating HSCAS into the diet enhanced cumulative BW gain, countered the rise in relative liver weight, partially mitigated adverse effects on serum biochemistry, and boosted the expression of catalase and superoxide dismutase in the liver for broiler chicks exposed to AFB1-contaminated feeds.

In addition to HSCASs, another promising natural adsorbent is discarded date pits (DDPs), a byproduct of the date fruit industry. They constitute approximately 10–15% of the total weight of the date fruit and are often discarded as waste [19]. The use of DDPs in poultry diets not only provides a cost-effective and sustainable solution for aflatoxin mitigation but also adds value to agriculture waste by-products as a dietary strategy in poultry nutrition [20]. It has been found that incorporating 10% DDPs can partially substitute dietary corn, improving gut health and promoting growth in broilers, potentially reducing production costs [20]. Date pits are rich in fiber, particularly insoluble fiber, which enhances their adsorptive capacity [21]. They also contain various bioactive compounds such as phenolic acids, flavonoids, and tannins, which have antioxidant and antimicrobial properties [22]. The high lignin and cellulose content in date pits further contribute to their potential as aflatoxin adsorbents by providing a complex matrix for toxin-binding [23]. In a recent study by Alharthi et al. [24], it was found that incorporating DDPs into broiler diets significantly alleviated the detrimental impacts of AFB1 on growth efficiency, carcass yield, liver health, intestinal integrity, blood biochemistry, and antioxidant capacity. Moreover, DDPs have been reported to effectively absorb aflatoxin M1 and ochratoxin A from contaminated milk, with removal rates of 56% and 52%, respectively, while inducing minimal alterations in nutritional milk constituents such as fat, protein, and lactose [25].

Despite such beneficial effects, there is a noteworthy lack of comprehensive studies that simultaneously assess the protective efficacy of HSCASs, a well-established mycotoxin binder, and DDPs, a novel natural feed additive, against the toxic effects induced by aflatoxins across a wide array of productive and physiological parameters in broiler chickens. Therefore, it is hypothesized that supplementing broiler diets with HSCASs and DDPs could mitigate the detrimental impacts of AFB1 on productive performance and physiological traits. In light of these hypotheses, this research aims to elucidate the extent to which dietary supplementation of HSCASs and DDPs can counteract the toxic effects of AFB1 and comprehensively assess their protective efficacy across various metrics, encompassing growth performance, carcass characteristics, serum biochemical indices, liver antioxidant status, hepatic function enzymes, ileum histological structure, apparent nutrient digestibility, and aflatoxin residues in the liver and kidney of broiler chickens exposed to aflatoxin-contaminated feed.

## 2. Materials and Methods

### 2.1. Ethical Approval

The animal study protocol was approved by the Ethics Committee of King Saud University, Riyadh, Saudi Arabia (KSU-SE-23-38).

### 2.2. Birds and Trial Design

In a controlled environment, 240 one-day-old mixed-sex Ross 308 broiler chicks of comparable initial body weight were housed in battery cages. Each cage accommodated six chicks, maintaining a density of 30 kg of body weight per m^2^. These chicks were fed a basal starter diet (Table 1) until they reached 15 days of age.

At 16 days of age, the cages were randomly assigned to one of four different dietary treatments. This assignment followed a completely randomized design, ensuring each dietary treatment had 10 replicates. The dietary treatments involved the following: the control group received the basal diet without AFB1 or any additives; the AFB1 group was given the basal diet contaminated with AFB1; the AFB1 + HSCAS group received the AFB1-contaminated diet supplemented with 0.5% HSCAS; and the AFB1 + DDP group was provided with the AFB1-contaminated diet supplemented with 4% DDP. The experiment was conducted over a period of 15 days. The AFB1 diet was crafted by substituting mycotoxin-free corn with naturally tainted corn, aiming to reach an AFB1 concentration of 1000 ppb, as formerly detailed by Yang et al. [26]. Employing a high-performance liquid chromatography system (Nexera XR, Shimadzu Corp., Kyoto, Japan), in accordance with procedures outlined by Peng et al. [27], the contaminated diet underwent testing for AFB1 and other kinds of aflatoxin. The resulting analysis demonstrated that the levels of other aflatoxins were below detectable thresholds. The HSCAS was added atop the mixture at a dosage determined and computed in accordance with the manufacturer’s guidelines. The Khalas variety of fresh DDP (*Phoenix dactylifera* L.) was sourced from Riyadh Dates Factory in Al Kharj, KSA. Subsequently, the DPs underwent grinding in a medium-sized mill (SK2500, Skiold A/S, Sæby, Denmark) to attain particles with a size of 1 mm.

Both starter (0–15 days) and finisher (16–30 days) corn–soybean meal-based diets, as detailed in Table 1, were crafted in mash form to fulfill the nutritional needs of Ross broiler chickens, aligning with the specifications provided by Aviagen [28]. During the entire period of the trial, chickens were provided with unfettered access to both feed and water, while being raised in accordance with the guidelines outlined in the Ross Broiler Management Handbook [29].

Throughout the 16- to 30-day period, calculations for feed intake (FI), body weight (BW), feed conversion rate corrected for mortality (FCR), and European production efficiency factor (EPEF) were conducted for each replication in order to assess performance metrics.

### 2.3. Sampling and Measurements

On day 26 of the experiment, ten birds from each treatment group were randomly chosen and placed in individual metabolic cages. Following a three-day adaptation period, the excreta generated by each bird were gathered over a 48-h span applying the total collection technique, as outlined by Alharthi et al. (2022) [24], while FI was simultaneously recorded. Afterward, the gathered feeds and excreta underwent oven-drying until reaching a consistent weight. They were then thoroughly ground to facilitate passage through a sieve with a mesh size of 0.5 mm. After the preparation procedure, chemical analyses were conducted on the specimens in accordance with the methods specified in AOAC [30] to quantify their crude protein (CP) content operating the Kjeldahl method (code 984.13) [30] and their ether extract (EE) content utilizing the Soxhlet extraction technique (code 920.39). The gross energy levels of the samplings were determined by employing a bomb calorimeter (IKA Calorimeter System C 5000; IKA Works Inc., Wilmington, NC, USA), calibrated with benzoic acid as the reference standard. The computation of the apparent digestibility of CP and EE, as well as the determination of the nitrogen-corrected apparent metabolizable energy (AMEn) in broilers, followed the methodology prescribed by De Marco et al. [31].

After the 30-day feeding trial concluded, 10 birds were randomly picked from each treatment group for sampling procedures. Blood samplings were assembled from the wing vein and then underwent centrifugation at 3000× *g* for 10 min at 4 °C to separate the serum, which was later analyzed biochemically. Commercial diagnostic kits (Randox Laboratories Ltd., Ardmore, Crumlin, UK) were utilized to quantify the serum levels of various indexes involving total protein (TP), albumin (ALB), glucose (GLU), alkaline phosphatase (ALP), and alanine aminotransferase (ALT), in accordance with the manufacturer’s guidelines. Additionally, serum globulin (GLO) concentrations were derived by subtracting ALB values from TP values.

After the blood specimens were assembled, the birds experienced several proceedings, encompassing weighing, euthanizing, plucking, processing, and evisceration. The dressing weight was determined by splitting the hot carcass weight by the pre-slaughter weight and was represented as a percentage. Furthermore, the weights of miscellaneous cut-up parts (breast muscles, leg quarters, and abdominal fat pads) and interior organs (liver, kidney, spleen, bursa of Fabricius, and empty gizzard) were taken and represented as a percentage relative to the pre-slaughter weight.

Roughly 0.5 g of liver tissues underwent homogenization in phosphate-buffered saline, followed by centrifugation at 3000× *g* for 10 min at 4 °C to gather supernatants for assessing antioxidative and functional activities. The content of protein in the gathered supernatants was assessed by employing the Bradford assay (Sigma-Aldrich, St. Louis, MO, USA). Levels of malondialdehyde (MDA), total antioxidant capacity (T-AOC), glutathione S-transferase (GST), glutathione peroxidase (GSH-Px), total superoxide dismutase (T-SOD), glutathione reductase (GR), glutamic oxaloacetic transaminase (GOT), and glutamic pyruvic transaminase (GPT) were quantified utilizing ELISA test kits (MyBioSource, San Diego, CA, USA) in accordance with the guidelines provided by the manufacturer. Afterward, all findings were adjusted relative to the total protein content in each specimen to facilitate comparisons across samples.

To prepare samples for histological analysis, 2 cm sections of the ileum from the midway area were washed with phosphate buffer saline and then put in a 10% neutral buffered formalin solution to fix them. Following fixation, the sections went through a series of steps comprising dehydration, clearing, embedding in paraffin wax, cutting into 5 μm thick slices, positioning on glass slides, and dyeing with hematoxylin and eosin, according to the procedures outlined by Williams et al. [32]. Villus height (VH) and width (VW) were determined utilizing ImageJ software, IJ 1.46r (National Institutes of Health, Bethesda, MD, USA) on at least 10 villi well-aligned within each cross-sectional slice. Subsequently, the villus surface area (VSA) was computed following the equation specified by Alqhtani et al. [33].

The process of isolating and purifying remaining AFB1 in liver and kidney tissues followed the previously described technique by Magnoli et al. [34]. Afterward, the identification and measurement of AFB1 in the resultant solution were conducted operating a high-performance liquid chromatography system with fluorescence detection (Nexera XR, Shimadzu Corp., Kyoto, Japan), pursuant to the methodology outlined by Cui et al. [35].

### 2.4. Statistical Analysis

Each bird served as the experimental unit, apart from performance measures where replication was regarded as the experimental unit. The gathered data underwent statistical examination through a one-way analysis of variance, complemented by Tukey’s test for conducting multiple comparisons, utilizing SAS software (version 9.4, SAS Institute Inc., Cary, NC, USA). Non-parametric data regarding the residues of AFB1 were evaluated utilizing the Kruskal–Wallis test, with mean comparisons performed via the post-hoc Dunn’s test. The threshold for significance was set at *p* < 0.05. The resulting values are proffered as least-square means along with their corresponding pooled standard error of the mean.

## 3. Results

### 3.1. Growth Performance

The impact of various dietary treatments on the growth performance of broiler chickens between 16 and 30 days of age is detailed in Table 2. When contrasted with the control group, broilers fed AFB1 exhibited a significant reduction in FI (*p* < 0.01), BW (*p* < 0.001), and EPEF (*p* < 0.001), along with an increased FCR (*p* < 0.001). Conversely, incorporating either HSCASs or DDPs into the AFB1-contaminated diet resulted in considerable enhancements in BW and reductions in FCR (*p* < 0.001), achieving levels comparable to those of the control group. However, the FI and EPEF values for the supplemented groups were intermediate and did not show significant differences from either the control or AFB1 groups.

### 3.2. Carcass Traits

The impact of various dietary treatments on carcass yields and visceral organ weights in 30-day-old broiler chickens is outlined in Table 3. In comparison to the control group, chickens consuming a diet contaminated with AFB1 exhibited significant reductions in dressing percentage (*p* < 0.001) and breast meat yield (*p* < 0.01), while experiencing augmentations in the proportional weights of the kidney (*p* < 0.05), liver (*p* < 0.001), and spleen (*p* < 0.05). Conversely, supplementing the AFB1-contaminated diet with either HSCASs or DDPs resulted in higher dressing proportions (*p* < 0.001) and lower relative weights of kidney and spleen (*p* < 0.05). The breast meat yield in these supplemented groups displayed no significant variances compared to both the control and AFB1 groups, placing it in an intermediate position between the two (*p* < 0.01). Moreover, the inclusion of DDPs in the AFB1 diet markedly diminished the relative liver weight compared to the AFB1 group (*p* < 0.001), with the liver weight for the AFB1 + HSCAS group lying between that of the AFB1 and AFB1 + DDP groups. Additionally, the relative weights of the kidney and liver in the AFB1 + DDP group were also intermediate, exhibiting no significant differences from those in the AFB1 + HSCAS or control groups. Across all dietary treatments, there were no significant changes observed in the relative weights of leg meat, abdominal fat, the bursa of Fabricius, or the empty gizzard (*p* > 0.05).

### 3.3. Blood Serum Indices

The impacts of different dietary treatments on the analysis of broiler serum, encompassing biochemical markers and liver function enzymes at the age of 30 days, are outlined in Table 4. In comparison to the control group, those consuming the AFB1-contaminated diet experienced considerable diminutions in the levels of TP (*p* < 0.001), ALB (*p* < 0.001), GLO (*p* < 0.01), and GLU (*p* < 0.001), alongside augmented activities of ALP (*p* < 0.01) and ALT (*p* < 0.05). On the other hand, adding HSCASs or DDPs to the diet contaminated with AFB1 significantly augmented levels of TP (*p* < 0.001), GLO (*p* < 0.01), and GLU (*p* < 0.001), while concurrently diminishing the activity of ALT (*p* < 0.05). The augmentations in GLO and GLU levels, along with a reduction in ALT activity brought on by these supplements, effectively mirrored those observed in the control group. Additionally, in comparison to the AFB1 group, the inclusion of DDP in the diet significantly heightened the ALB concentration (*p* < 0.001) and lowered ALP activity (*p* < 0.01). This reduction in ALP activity brought levels comparable to those observed in the control group. However, in the AFB1 + HSCAS group, the concentration of ALB fell between that of the AFB1 and AFB1 + DDP groups, showing no significant difference from either. Meanwhile, the activity of ALP in this group was also an intermediary, lying between the levels observed in the other treatment groups.

### 3.4. Liver Antioxidant Capacity and Function Enzymes

The influence of various dietary treatments on the oxidative status and functional enzymes in the livers of 30-day-old broiler chickens is displayed in Table 5. In contrast to the control group, feeding AFB1 led to diminished levels of protein (*p* < 0.001), T-AOC (*p* < 0.001), GSH-Px (*p* < 0.01), and T-SOD (*p* < 0.05), alongside heightened MDA content (*p* < 0.001) and GOT activity (*p* < 0.05). In contrast, supplementing the AFB1-contaminated diet with either HSCASs or DDPs resulted in elevated protein levels (*p* < 0.001) and GSH-Px activity (*p* < 0.01), accompanied by a concurrent reduction in MDA content (*p* < 0.001) and GOT activity (*p* < 0.05). The MDA content for the AFB1 + HSCAS group fell between that of the control and AFB1 + DDP groups, indicating an intermediary effect. Considerably, the rise in GSH-Px activity resulting from DDP supplementation surpassed that of the AFB1 + HSCAS group. Moreover, adding DDPs to the diet raised the T-AOC concentration to levels comparable to the control group (*p* < 0.001), exceeding levels in the AFB1 and AFB1 + HSCAS groups. Additionally, the activity of T-SOD in the supplemented groups was intermediate, falling between the control and AFB1 groups. However, no variations were observed in the activities of GST, GR, and GPT across the treatment groups (*p* > 0.05).

### 3.5. Ileal Morphology, Nutrient Digestibility, and AFB1 Residues

The influences of different dietary treatments on ileal histomorphometry, apparent nutrient digestibility, and the concentrations of AFB1 residues in the liver and kidney tissues of 30-day-old broiler chickens are illustrated in Table 6. When contrasted with the control, chickens fed the AFB1-contaminated diet showed significant reductions in VH (*p* < 0.001), VW (*p* < 0.01), VSA (*p* < 0.05), and the digestibility of CP (*p* < 0.001), EE (*p* < 0.05), and AMEn (*p* < 0.001). Moreover, these chickens had higher levels of AFB1 residues in both liver and kidney tissues, while the control group displayed no detectable residues (*p* < 0.001). On the other hand, supplementing the contaminated diet with HSCASs or DDPs led to significant improvements in VH (*p* < 0.001), VSA (*p* < 0.05), and AMEn (*p* < 0.001), along with the diminished accumulation of AFB1 residues in both liver and kidney tissues (*p* < 0.001). Additionally, the VW of the AFB1 + DDP group and the CP digestibility of the AFB1 + HSCAS group reached levels akin to those observed in the control group. However, the VH, VSA, and AMEn parameters for the AFB1 + DDP group exhibited intermediate values between those of the control group and those observed in chickens treated with AFB1 and HSCAS. Likewise, the AFB1 + HSCAS group exhibited intermediate VW levels, while the AFB1 + DDP group showcased intermediary CP digestibility, without significant deviations from the other treatments (*p* < 0.01). Additionally, the EE digestibility in the supplemented groups fell between those observed in the control and AFB1 groups.

## 4. Discussion

The current study highlights the adverse impact of AFB1 contamination on broiler productivity and health, while also assessing the effectiveness of HSCASs and DDPs as potential mitigation agents. The significant reductions in FI, BW, and EPEF, coupled with an increased FCR, underscore the potent toxicity of AFB1. These findings are consistent with previous studies reporting growth retardation and impaired feed conversion in broilers fed AFB1-contaminated diets [36,37]. The decreased FI observed in AFB1-fed broilers can be attributed to the toxic effects of AFB1 on the liver, which plays a crucial role in metabolism and appetite regulation. AFB1-induced liver damage likely leads to anorexia and reduced nutrient absorption and utilization, ultimately resulting in lower BW and higher FCR [38,39]. The significant reduction in EPEF further underscores the negative impact of AFB1 on overall production efficiency, as this factor combines growth rate, feed efficiency, and survivability into a single metric. Encouragingly, incorporating HSCAS or DDP into the AFB1-contaminated diet resulted in substantial improvements in BW and reductions in FCR, bringing these metrics to levels comparable to those of the control group. Our findings are consistent with previous research demonstrating the efficacy of HSCAS supplementation in alleviating the adverse effects of AFB1 on broiler growth performance [40]. Our results also align with a recent study that has highlighted the potential of date pits degraded via the cellulolytic fungus *Trichoderma reesei* in enhancing broiler gut health and growth performance [20].

Chickens consuming the AFB1-contaminated diet exhibited significant reductions in dressing percentage and breast meat yield, alongside significant increases in the proportional weights of the kidney, liver, and spleen compared to the control group. These findings are in line with prior research showing that AFB1 toxicity adversely impacts both carcass quality and visceral organ weights in broiler chickens [41]. AFB1 is known to impair protein synthesis and disrupt metabolic processes, leading to reduced muscle development and overall carcass yield [42]. The increased relative weights of the kidney, liver, and spleen suggest a compensatory response to the toxin’s damaging effects. The liver, being the primary site of aflatoxin metabolism, is often enlarged due to hepatic cell proliferation in response to the damage caused by AFB1 [42]. Similarly, the increased spleen weight could indicate an immune response to the toxin, while kidney enlargement may reflect the organ’s attempt to excrete AFB1 metabolites [43]. Promisingly, our results demonstrated that supplementing the AFB1-contaminated diet with HSCASs significantly improved dressing percentage and reduced the relative weights of the kidney and spleen. The results of this study align with those of previous research, which has demonstrated the efficacy of HSCASs in mitigating the adverse effects of AFB1 on broiler health and productivity [44]. The inclusion of DDPs in the AFB1 diet yielded even more promising results. Not only did it enhance dressing percentage and reduce relative kidney and spleen weights but it also markedly diminished the relative liver weight compared to both the AFB1 and AFB1 + HSCAS groups. Our findings align with a recent study that also underscored the beneficial effects of date pits in enhancing carcass traits and visceral organ development of broilers under AFB1 stress [24].

Our findings reveal that AFB1 exposure significantly disrupts broiler health, marked by decreased serum levels of TP, ALB, GLO, and GLU, alongside elevated ALP and ALT serum activities. These findings align with previous studies on broilers indicating that AFB1 disrupts protein synthesis and glucose metabolism, leading to hypoproteinemia and hypoglycemia due to liver damage [45]. The observed decrease in TP, ALB, and GLO levels in the AFB1 group can be attributed to the toxin’s detrimental impact on protein synthesis and metabolism in the liver [46]. The reduction in GLU levels may result from impaired gluconeogenesis and glycogenolysis, vital processes for maintaining glucose homeostasis [46]. The observed elevation in ALP and ALT activities is consistent with prior research, indicating heightened serum liver enzyme levels as biomarkers of hepatocellular injury in broilers exposed to AFB1 [47]. The elevated ALP and ALT activities in the AFB1 group could be ascribed to hepatic damage and dysfunction, as these enzymes are released into the bloodstream in response to hepatocellular damage and cholestasis [48]. The addition of HSCAS and DDP to the AFB1-contaminated diet significantly ameliorated the adverse effects of AFB1 on broiler serum biochemistry. The considerable increase in TP, GLO, and GLU levels in the HSCAS and DDP groups suggests the mitigation of AFB1’s toxic effects on protein synthesis and carbohydrate metabolism. The increase in GLO levels, in particular, indicates a positive impact on the immune response, as globulins play a crucial role in antibody production [47]. The reduction in ALT activity further indicates a protective effect on hepatocytes. Interestingly, DDP exhibited superior efficacy compared to HSCAS, as evidenced by the significantly higher ALB level and lower ALP activity in the DDP group. This suggests that DDP might be more effective in protecting against AFB1-induced liver damage. Our findings corroborate recent studies demonstrating the efficacy of HSCAS and DDP as feed supplements in mitigating the adverse effects of AFB1 on broiler health, particularly by attenuating liver damage and improving metabolic function [24,49].

The findings of the current study demonstrate the deleterious effects of AFB1 on liver health, as evidenced by reduced total protein levels, diminished activities of T-AOC, GSH-Px, and T-SOD, elevated MDA content as a hallmark of oxidative stress, and heightened GOT activity. These findings align with a broad body of literature demonstrating that AFB1 disrupts protein synthesis and metabolism, impairs antioxidant defense mechanisms, induces oxidative damage, and triggers lipid peroxidation in poultry liver, leading to hepatocyte damage, liver dysfunction, and cellular enzyme leakage [38,50]. In contrast, supplementing the AFB1-contaminated diet with HSCAS partially mitigated the toxic influences of AFB1 on broiler livers, as demonstrated by augmented protein levels and GSH-Px activity, along with reduced MDA content and GOT activity in hepatic tissue. This finding is in line with previous research reporting the protective influences of HSCASs against AFB1-induced oxidative stress and hepatic dysfunction in broiler chicks [18]. The addition of DDPs to the AFB1-contaminated diet exhibited a more pronounced protective effect compared to HSCASs. DDPs not only enhanced protein levels and GSH-Px activity while reducing MDA content and GOT activity but also significantly raised the T-AOC concentration compared to both the AFB1 and AFB1 + HSCAS groups, reaching levels comparable to those of the control group. Furthermore, it raised GSH-Px activity to a level exceeding that of the AFB1 + HSCAS group. This suggests that DDPs are likely a more potent agent than HSCASs in mitigating AFB1-induced liver damage. Our findings are consistent with a recent study on broiler chickens highlighting the potential of DDPs as a natural feed additive for effectively mitigating AFB1-induced hepatotoxicity [51].

Our results revealed that AFB1 contamination markedly compromised intestinal morphology, resulting in decreased villus height, width, and surface area in the ileum of broilers, likely due to necrosis and shedding of enterocytes [52]. These alterations in the ultrastructure of the ileal mucosa likely contributed to the reductions observed in the apparent digestibility of CP, EE, and AMEn in the AFB1 group. These results are consistent with previous studies that have reported similar deleterious effects of AFB1 on intestinal morphology and nutrient utilization in broilers [53]. Additionally, the accumulation of AFB1 residues in liver and kidney tissues further confirms the toxicokinetic behavior of AFB1, where these organs are primary sites of biotransformation and excretion [54]. These findings support earlier research documenting the bioaccumulation of AFB1 and its metabolites in various organs of broilers [53]. Supplementing the AFB1-contaminated diet with HSCASs led to significant improvements in VH and VSA, which potentially contributed to the observed enhancement in CP and AMEn digestibility in the AFB1 + HSCAS group, suggesting better nutrient absorption and utilization that could boost growth performance in broilers. Interestingly, adding DDPs to the AFB1-contaminated diet not only enhanced VH and VSA but also restored VW to a level comparable to that of the control group, indicating a significant restoration of intestinal lining integrity. While DDPs did not match HSCASs in enhancing CP digestibility, they effectively improved AMEn digestibility, suggesting a potentially stronger impact on energy utilization and possibly influencing the metabolism of fats and carbohydrates. Furthermore, our study showcased the effectiveness of HSCASs and DDPs in decreasing AFB1 residues in liver and kidney tissues, highlighting their potential as powerful agents for mycotoxin detoxification. These findings align with a recent study that showed that adding HSCASs to AFB1-contaminated feed partially prevented aflatoxicosis by reducing aflatoxin residues in the liver and kidneys of broiler chicks [55]. The findings of this study also agree with recent research reporting the efficacy of dietary DDPs in mitigating the adverse effects of AFB1 exposure on ileal morphology, nutrient digestibility, and hepatic AFB1 residues in broiler chickens [24]. The restoration of VW in the AFB1 + DDP group and CP digestibility in the AFB1 + HSCAS group to control levels suggests that these adsorbents may offer targeted protection against specific aspects of AFB1-induced damage, likely due to their distinct chemical compositions and binding properties. However, additional research is necessary to uncover the precise mechanisms responsible for these differing effects.

The protective effects of HSCAS against AFB1 toxicity are likely due to its high affinity for binding aflatoxins within its porous structure in the gastrointestinal tract, thereby preventing the toxin from being absorbed into the bloodstream and subsequently transported to vital organs such as the liver [56]. By adsorbing and sequestering AFB1, HSCASs reduce its bioavailability, mitigating its systemic toxic effects and hepatotoxicity [57]. This mechanism also contributes to the enhanced excretion of AFB1 or its metabolites [58], resulting in lower residue levels and potentially explaining the observed improvements in productive performance and health status in HSCAS-supplemented broilers. The use of DDPs as a dietary supplement is relatively novel, and our study contributes to the growing body of evidence supporting their efficacy in mitigating the effects of aflatoxins. The effectiveness of DDPs is attributed to their high fiber content and the presence of bioactive compounds, such as polyphenols, phenolic acids, and flavonoids, which possess antioxidant and anti-inflammatory properties [59]. These properties potentially aid in reducing oxidative stress and liver damage caused by AFB1, thereby improving nutrient absorption and growth performance in broilers. The fibrous nature of DDPs, rich in cellulose and lignin, may bind AFB1 in the gut, preventing its uptake into the bloodstream and systemic distribution [60], thereby contributing to the observed reduction in liver weight and damage. The superior efficacy of DDPs compared to HSCASs may be due to their ability to scavenge free radicals, inhibit lipid peroxidation, and enhance the liver’s antioxidant defense system, thus protecting against AFB1-induced oxidative stress [61]. Additionally, DDPs may facilitate the repair and regeneration of intestinal epithelium by preventing AFB1’s interaction with intestinal cells and modulating intestinal microbiota [25]. Further research is required to elucidate the specific mechanisms underlying DDPs’ hepatoprotective effects and their potential use in poultry feed to combat aflatoxicosis.

## 5. Conclusions

The findings of this study demonstrate that including HSCASs and DDPs in the AFB1-contaminated diet significantly mitigated its toxic effects on the health and performance of broiler chickens aged 16 to 30 days. Specifically, incorporating these dietary adsorbents resulted in increased BW, improved FCR, enhanced dressing percentage, reduced relative weights of kidney and spleen, elevated serum levels of TP, GLO, and GLU, lowered serum ALT activity, augmented hepatic protein concentration and GSH-Px activity, and reduced hepatic MDA content and GOT activity. Additionally, they enhanced ileal VH and VSA, improved AMEn digestibility, and reduced AFB1 residues in the liver and kidney tissues. Interestingly, DDP supplementation showed superior efficacy over HSCAS by significantly reducing relative liver weight, raising serum ALB concentration, lowering serum ALP activity, enhancing hepatic T-AOC level, and increasing ileal VW. Conversely, HSCAS addition was particularly effective in improving apparent CP digestibility. These results underscore the potential of HSCASs and DDPs as effective dietary strategies to mitigate the adverse effects of AFB1 contamination in broiler chickens, with DDPs showing superior efficacy in several health metrics.

## Figures and Tables

**Table 1:** The constituents and nutritional values (%, as-fed basis, unless stated otherwise) of the starter (0–15 days) and finisher (16–30 days) diets.

Ingredients	Starter Diet 0–15 d	Control Diet 16–30 d	DDP Diet 16–30 d
Yellow corn	54.2	59.8	56.0
Soybean meal	31.4	27.0	25.0
Corn gluten meal	7.30	5.10	6.60
DDP ^1^	0.00	0.00	4.00
Palm oil	2.20	4.14	4.46
Dicalcium phosphate	1.88	1.68	1.68
Ground limestone	1.10	0.87	0.87
Salt	0.40	0.40	0.40
DL-methionine	0.30	0.26	0.26
Lysine-HCL	0.40	0.26	0.33
Threonine	0.14	0.08	0.10
Vitamin and mineral premix ^2^	0.20	0.20	0.20
Total	100	100	100
Analyzed nutrients			
Metabolizable energy (kcal/kg)	3025	3114	3110
Crude protein	23.1	19.94	19.95
Available phosphorus	0.48	0.41	0.41
Calcium	0.96	0.81	0.81
Digestible lysine	1.27	1.05	1.05
Digestible sulfur amino acids	0.96	0.85	0.85
Digestible threonine	0.87	0.70	0.70

^1^ DDP, discarded date pits. ^2^ The vitamin–mineral premix delivered the following content per kg of diet: retinol, 12,000,000 IU; cholecalciferol, 5,000,000 IU; tocopherol, 80,000 IU; menadione, 3200 mg; thiamine, 3200 mg; riboflavin, 8600 mg; niacin, 65,000 mg; pantothenic acid, 20,000 mg; pyridoxine, 4300 mg; biotin, 220 mg; folic acid, 2200 mg; cyanocobalamin, 17 mg; antioxidants (butylated hydroxyanisole and butylated hydroxytoluene), 50,000 mg; copper, 16,000 mg; iodine, 1250 mg; iron, 20,000 mg; manganese, 120,000 mg; selenium, 300 mg; zinc, 110,000 mg.

**Table 2:** Impact of adding hydrated sodium calcium aluminosilicates (HSCASs) and discarded date pits (DDPs) to aflatoxin B1 (AFB1)-contaminated diet on the growth performance of broilers aged 16–30 days.

Items *	FI (g)	BW (g)	FCR (g:g)	EPEF (%)
Control	1862 ^a^	1315 ^a^	1.416 ^b^	363 ^a^
AFB1	1732 ^b^	1073 ^b^	1.614 ^a^	291 ^b^
AFB1 + HSCAS	1844 ^ab^	1280 ^a^	1.440 ^b^	329 ^ab^
AFB1 + DDP	1845 ^ab^	1274 ^a^	1.448 ^b^	330 ^ab^
SEM	30.2	26.2	0.021	7.11
*p*-value	0.01	0.001	0.001	0.001

^a,b^ Values in the same column marked by different superscripts indicate significant differences (*p* < 0.05). * FI, feed intake; BW, body weight; FCR, feed conversion ratio; EPEF, European production efficiency factor; SEM, pooled standard error of the mean.

**Table 3:** Impact of adding hydrated sodium calcium aluminosilicates (HSCASs) and discarded date pits (DDPs) to aflatoxin B1 (AFB1)-contaminated diet on the relative weights (% of pre-slaughter weight) of carcass yields and visceral organs in broilers at 30 days.

Items *	Dressing	Breast	Leg	Fat	Kidney	Liver	Bursa	Spleen	Gizzard
Control	71.3 ^a^	27.8 ^a^	20.8	1.05	0.62 ^c^	2.01 ^c^	0.17	0.13 ^b^	1.82
AFB_1_	69.6 ^c^	25.4 ^b^	20.2	1.14	1.05 ^a^	3.42 ^a^	0.17	0.25 ^a^	2.15
AFB1 + HSCAS	70.7 ^b^	26.7 ^ab^	20.4	1.07	0.84 ^b^	2.75 ^ab^	0.17	0.16 ^b^	2.10
AFB1 + DDP	70.8 ^b^	26.9 ^ab^	20.9	0.92	0.71 ^bc^	2.37 ^bc^	0.18	0.14 ^b^	1.99
SEM	0.33	0.61	0.23	0.14	0.02	0.09	0.011	0.015	0.15
*p*-value	0.001	0.01	0.256	0.325	0.05	0.001	0.906	0.05	0.158

^a–c^ Values in the same column marked by different superscripts indicate significant differences (*p* < 0.05). * SEM, pooled standard error of the mean (*n* = 10).

**Table 4:** Impact of adding hydrated sodium calcium aluminosilicates (HSCASs) and discarded date pits (DDPs) to aflatoxin B1 (AFB1)-contaminated diet on the blood serum indicators of broilers at 30 days.

Items *	Biochemical Indices				Liver Function Enzymes	
	TP	ALB	GLO	GLU	ALP	ALT
	g/dL	g/dL	g/dL	mg/dL	U/L	U/L
Control	5.15 ^a^	2.52 ^a^	2.63 ^a^	213 ^a^	264 ^b^	5.2 ^b^
AFB1	3.93 ^c^	1.98 ^c^	1.95 ^b^	167 ^c^	374 ^a^	12.7 ^a^
AFB1 + HSCAS	4.81 ^b^	2.13 ^bc^	2.68 ^a^	206 ^a^	314 ^ab^	7.9 ^b^
AFB1 + DDP	4.92 ^b^	2.33 ^b^	2.59 ^a^	201 ^a^	281 ^b^	6.7 ^b^
SEM	0.092	0.051	0.084	3.01	11.2	0.54
*p*-value	0.001	0.001	0.01	0.001	0.01	0.05

^a–c^ Values in the same column marked by different superscripts indicate significant differences (*p* < 0.05). * TP, total protein; ALB, albumin; GLO, globulin; GLU, glucose; ALP, alkaline phosphatase; ALT, alanine aminotransferase; SEM, pooled standard error of the mean (*n* = 10).

**Table 5:** Impact of adding hydrated sodium calcium aluminosilicates (HSCASs) and discarded date pits (DDPs) to aflatoxin B1 (AFB1)-contaminated diet on the hepatic antioxidant capacity and function enzymes of broilers at 30 days.

Items *		Antioxidant Capacity						Function Enzymes	
	Protein	MDA	T-AOC	GST	GSH-Px	T-SOD	GR	GOT	GPT
	mg/100 mg Tissue	nmol/mg Protein	U/mg of Protein						
Control	9.21 ^a^	0.34 ^c^	2.17 ^a^	32.1	50.5 ^a^	235 ^a^	6.80	53.2 ^b^	8.21
AFB1	6.12 ^c^	0.61 ^a^	1.67 ^b^	33.1	43.5 ^d^	201 ^b^	6.08	55.8 ^a^	9.98
AFB1 + HSCAS	8.05 ^b^	0.38 ^bc^	1.78 ^b^	33.5	45.1 ^c^	217 ^ab^	6.47	54.3 ^b^	9.07
AFB1 + DDP	8.15 ^b^	0.43 ^b^	2.05 ^a^	33.5	47.8 ^b^	215 ^ab^	6.22	54.1 ^b^	8.98
SEM	0.084	0.015	0.078	0.44	0.33	2.12	0.21	0.73	0.54
*p*-value	0.001	0.001	0.001	0.211	0.01	0.05	0.245	0.05	0.12

^a–d^ Values in the same column marked by different superscripts indicate significant differences (*p* < 0.05). * MDA, malondialdehyde; T-AOC, total antioxidant capacity; GST, glutathione S-transferase; GSH-Px, glutathione peroxidase; T-SOD, total superoxide dismutase; GR, glutathione reductase; GOT, glutamic oxaloacetic transaminase; GPT, glutamic pyruvic transaminase; SEM, pooled standard error of the mean (*n* = 10).

**Table 6:** Impact of adding hydrated sodium calcium aluminosilicates (HSCASs) and discarded date pits (DDPs) to aflatoxin B1 (AFB1)-contaminated diet on the ileal histology, apparent nutrient digestibility, and residual AFB1 levels in the liver and kidney of broilers at 30 days.

Items ^1^	Ileal Histology			Nutrient Digestibility			AFB1 Residues ^2^	
	VH	VW	VSA	CP	EE	AMEn	Liver	Kidney
	µm	µm	μm^2^	%	%	kcal/kg	µg/kg	
Control	641 ^a^	67.5 ^a^	0.14 ^a^	64.2 ^a^	78.9 ^a^	3175 ^a^	ND	ND
AFB1	545 ^c^	62.1 ^b^	0.11 ^c^	56.2 ^b^	73.2 ^b^	3056 ^c^	4.92 ^a^	1.21 ^a^
AFB1 + HSCAS	588 ^b^	64.3 ^ab^	0.12 ^b^	62.5 ^a^	76.9 ^ab^	3108 ^b^	1.54 ^b^	0.29 ^b^
AFB1 + DDP	618 ^ab^	66.5 ^a^	0.13 ^ab^	62.2 ^ab^	76.8 ^ab^	3148 ^ab^	1.45 ^b^	0.35 ^b^
SEM	22.2	3.77	0.006	0.92	1.56	10.23	0.12	0.065
*p*-value	0.001	0.01	0.05	0.001	0.05	0.001	0.001	0.001

^a–c^ Values in the same column marked by different superscripts indicate significant differences (*p* < 0.05). ^1^ VH, villus height; VW, villus width; VSA, villus surface area; CP, crude protein; EE, ether extract; AMEn, nitrogen-corrected apparent metabolizable energy; SEM, pooled standard error of the mean (*n* = 10). ^2^ The data on AFB1 residues were analyzed using the Kruskal–Wallis test; ND, not detectable.

## Data Availability

The raw data supporting the conclusions of this article will be made available by the authors upon request.

## References

[1] Fouad A.M., Ruan D., El Senousey H.A.K., Chen W., Jiang S., Zheng C. (2019). Harmful Effects and Control Strategies of Aflatoxin B1 Produced by Aspergillus Flavus and Aspergillus Parasiticus Strains on Poultry: Review. Toxins.

[2] Haque M.A., Wang Y., Shen Z., Li X., Saleemi M.K., He C. (2020). Mycotoxin Contamination and Control Strategy in Human, Domestic Animal and Poultry: A Review. Microb. Pathog..

[3] Umaya S.R., Vijayalakshmi Y.C., Sejian V. (2021). Exploration of Plant Products and Phytochemicals against Aflatoxin Toxicity in Broiler Chicken Production: Present Status. Toxicon.

[4] Gao S., Zhang L., Zhu D., Huang J., Yang J., Jiang J., Wu H., Lv G. (2022). Effects of Glucose Oxidase and Bacillus Subtilis on Growth Performance and Serum Biochemical Indicexs of Broilers Exposed to Aflatoxin B1 and Endotoxin. Anim. Feed Sci. Technol..

[5] Zou Y., Liu S.B., Zhang Q., Tan H.Z. (2023). Effects of Aflatoxin B1 on Growth Performance, Carcass Traits, Organ Index, Blood Biochemistry and Oxidative Status in Chinese Yellow Chickens. J. Vet. Med. Sci..

[6] Tavangar P., Gharahveysi S., Rezaeipour V., Irani M. (2021). Efficacy of Phytobiotic and Toxin Binder Feed Additives Individually or in Combination on the Growth Performance, Blood Biochemical Parameters, Intestinal Morphology, and Microbial Population in Broiler Chickens Exposed to Aflatoxin B1. Trop. Anim. Health Prod..

[7] Muhammad I., Wang H., Sun X., Wang X., Han M., Lu Z., Cheng P., Hussain M.A., Zhang X. (2018). Dual Role of Dietary Curcumin through Attenuating AFB1-Induced Oxidative Stress and Liver Injury via Modulating Liver Phase-I and Phase-II Enzymes Involved in AFB1 Bioactivation and Detoxification. Front Pharmacol..

[8] Rashidi N., Khatibjoo A., Taherpour K., Akbari-Gharaei M., Shirzadi H. (2020). Effects of Licorice Extract, Probiotic, Toxin Binder and Poultry Litter Biochar on Performance, Immune Function, Blood Indices and Liver Histopathology of Broilers Exposed to Aflatoxin-B1. Poult. Sci..

[9] Sarker M.T., Wang Z.Y., Yang H., Wan X., Emmanuel A. (2021). Evaluation of the Protective Effect of Lycopene on Growth Performance, Intestinal Morphology, and Digestive Enzyme Activities of AflatoxinB1 Challenged Broilers. Anim. Sci..

[10] Liu N., Ding K., Wang J., Deng Q., Gu K., Wang J. (2018). Effects of Lactic Acid Bacteria and Smectite after Aflatoxin B1 Challenge on the Growth Performance, Nutrient Digestibility and Blood Parameters of Broilers. J. Anim. Physiol. Anim. Nutr..

[11] Guo H., Wang P., Liu C., Chang J., Yin Q., Wang L., Jin S., Zhu Q., Lu F. (2023). Compound Mycotoxin Detoxifier Alleviating Aflatoxin B1 Toxic Effects on Broiler Growth Performance, Organ Damage and Gut Microbiota. Poult. Sci..

[12] Putra R.P., Astuti D., Respati A.N., Ningsih N., Triswanto, Yano A.A., Gading B.M.W.T., Jayanegara A., Sholikin M.M., Hassim H.A. (2024). Protective Effects of Feed Additives on Broiler Chickens Exposed to Aflatoxins-Contaminated Feed: A Systematic Review and Meta-Analysis. Vet. Res. Commun..

[13] Liu M., Zhao L., Gong G., Zhang L., Shi L., Dai J., Han Y., Wu Y., Khalil M.M., Sun L. (2022). Invited Review: Remediation Strategies for Mycotoxin Control in Feed. J. Anim. Sci. Biotechnol..

[14] Elliott C.T., Connolly L., Kolawole O. (2020). Potential Adverse Effects on Animal Health and Performance Caused by the Addition of Mineral Adsorbents to Feeds to Reduce Mycotoxin Exposure. Mycotoxin Res..

[15] Kihal A., Rodríguez-Prado M., Calsamiglia S. (2022). The Efficacy of Mycotoxin Binders to Control Mycotoxins in Feeds and the Potential Risk of Interactions with Nutrient: A Review. J. Anim. Sci..

[16] Peng Z., Chen L., Zhu Y., Huang Y., Hu X., Wu Q., Nüssler A.K., Liu L., Yang W. (2018). Current Major Degradation Methods for Aflatoxins: A Review. Trends Food Sci. Technol..

[17] Yiannikouris A., Apajalahti J., Kettunen H., Ojanperä S., Bell A.N.W., Keegan J.D., Moran C.A. (2021). Efficient Aflatoxin B1 Sequestration by Yeast Cell Wall Extract and Hydrated Sodium Calcium Aluminosilicate Evaluated Using a Multimodal In-Vitro and Ex-Vivo Methodology. Toxins.

[18] Chen X., Horn N., Applegate T.J. (2014). Efficiency of Hydrated Sodium Calcium Aluminosilicate to Ameliorate the Adverse Effects of Graded Levels of Aflatoxin B1 in Broiler Chicks. Poult. Sci..

[19] Attia A.I., Reda F.M., Patra A.K., Elnesr S.S., Attia Y.A., Alagawany M. (2021). Date (*Phoenix dactylifera* L.) by-Products: Chemical Composition, Nutritive Value and Applications in Poultry Nutrition, an Updating Review. Animals.

[20] Alyileili S.R., Belal I.E.H., Hussein A.S., El-Tarabily K.A. (2020). Effect of Inclusion of Degraded and Non-Degraded Date Pits in Broilers’ Diet on Their Intestinal Microbiota and Growth Performance. Animals.

[21] Abdelnaby A., Abdelaleem N.M., Elshewy E., Mansour A.H., Ibrahim S.S. (2023). Application of Bentonite Clay, Date Pit, and Chitosan Nanoparticles as Promising Adsorbents to Sequester Toxic Lead and Cadmium from Milk. Biol. Trace. Elem. Res..

[22] Liu Y., Wei S., Wu M., Yang S. (2018). Phenolic Compounds from Date Pits: Ultrasonic-Assisted Extraction, Antioxidant Activity and Component Identification. J. Food Meas. Charact..

[23] El-Far A.H., Ahmed H.A., Shaheen H.M. (2016). Dietary Supplementation of Phoenix Dactylifera Seeds Enhances Performance, Immune Response, and Antioxidant Status in Broilers. Oxid. Med. Cell. Longev..

[24] Alharthi A.S., Al Sulaiman A.R., Aljumaah R.S., Alabdullatif A.A., Elolimy A.A., Alqhtani A.H., Al-Garadi M.A., Abudabos A.M. (2022). Protective Effect of Date Pits on Growth Performance, Carcass Traits, Blood Indices, Intestinal Morphology, Nutrient Digestibility, and Hepatic Aflatoxin Residues of Aflatoxin B1-Exposed Broilers. Agriculture.

[25] Abdelnaby A., Abdelaleem N.M., Elshewy E., Mansour A.H., Ibrahim S. (2022). The Efficacy of Clay Bentonite, Date Pit, and Chitosan Nanoparticles in the Detoxification of Aflatoxin M1 and Ochratoxin A from Milk. Environ. Sci. Pollut. Res..

[26] Yang J., Bai F., Zhang K., Bai S., Peng X., Ding X., Li Y., Zhang J., Zhao L. (2012). Effects of Feeding Corn Naturally Contaminated with Aflatoxin B1 and B2 on Hepatic Functions of Broilers. Poult. Sci..

[27] Peng H., Chang Y., Baker R.C., Zhang G. (2019). Interference of Mycotoxin Binders with ELISA, HPLC and LC-MS/MS Analysis of Aflatoxins in Maize and Maize Gluten. Food Addit. Contam. Part A.

[28] Aviagen Group (2022). Ross Broiler: Nutrition Specifications.

[29] Aviagen Group (2018). Ross Broiler: Management Handbook.

[30] AOAC International (2019). Official Methods of Analysis of the Association of Analytical Chemists.

[31] De Marco M., Martínez S., Hernandez F., Madrid J., Gai F., Rotolo L., Belforti M., Bergero D., Katz H., Dabbou S. (2015). Nutritional Value of Two Insect Larval Meals (*Tenebrio molitor* and *Hermetia illucens*) for Broiler Chickens: Apparent Nutrient Digestibility, Apparent Ileal Amino Acid Digestibility and Apparent Metabolizable Energy. Anim. Feed. Sci. Technol..

[32] Williams J.M., Duckworth C.A., Vowell K., Burkitt M.D., Pritchard D.M. (2016). Intestinal Preparation Techniques for Histological Analysis in the Mouse. Curr. Protoc. Mouse Biol..

[33] Alqhtani A.H., Al Sulaiman A.R., Alharthi A.S., Abudabos A.M. (2022). Effect of Exogenous Enzymes Cocktail on Performance, Carcass Traits, Biochemical Metabolites, Intestinal Morphology, and Nutrient Digestibility of Broilers Fed Normal and Low-Energy Corn–Soybean Diets. Animals.

[34] Magnoli A.P., Monge M.P., Miazzo R.D., Cavaglieri L.R., Magnoli C.E., Merkis C.I., Cristofolini A.L., Dalcero A.M., Chiacchiera S.M. (2011). Effect of Low Levels of Aflatoxin B1 on Performance, Biochemical Parameters, and Aflatoxin B1 in Broiler Liver Tissues in the Presence of Monensin and Sodium Bentonite. Poult. Sci..

[35] Cui X., Muhammad I., Li R., Jin H., Guo Z., Yang Y., Hamid S., Li J., Cheng P., Zhang X. (2017). Development of a UPLC-FLD Method for Detection of Aflatoxin B1 and M1 in Animal Tissue to Study the Effect of Curcumin on Mycotoxin Clearance Rates. Front. Pharmacol..

[36] Nazarizadeh H., Pourreza J. (2019). Evaluation of Three Mycotoxin Binders to Prevent the Adverse Effects of Aflatoxin B1 in Growing Broilers. J. Appl. Anim. Res..

[37] Lai Y., Sun M., He Y., Lei J., Han Y., Wu Y., Bai D., Guo Y., Zhang B. (2022). Mycotoxins Binder Supplementation Alleviates Aflatoxin B1 Toxic Effects on the Immune Response and Intestinal Barrier Function in Broilers. Poult. Sci..

[38] Wang Y., Wang X., Li Q. (2023). Aflatoxin B1 in Poultry Liver: Toxic Mechanism. Toxicon.

[39] Mughal M.J., Peng X., Kamboh A.A., Zhou Y., Fang J. (2017). Aflatoxin B1 Induced Systemic Toxicity in Poultry and Rescue Effects of Selenium and Zinc. Biol. Trace Elem. Res..

[40] Liu N., Wang J., Deng Q., Gu K., Wang J. (2018). Detoxification of Aflatoxin B1 by Lactic Acid Bacteria and Hydrated Sodium Calcium Aluminosilicate in Broiler Chickens. Livest. Sci..

[41] Alharthi A.S., Al Sulaiman A.R., Aljumaah R.S., Alabdullatif A.A., Ferronato G., Alqhtani A.H., Al-Garadi M.A., Al-sornokh H., Abudabos A.M. (2022). The Efficacy of Bentonite and Zeolite in Reducing Aflatoxin B1 Toxicity on Production Performance and Intestinal and Hepatic Health of Broiler Chickens. Ital. J. Anim. Sci..

[42] Monson M.S., Coulombe R.A., Reed K.M. (2015). Aflatoxicosis: Lessons from Toxicity and Responses to Aflatoxin B1 in Poultry. Agriculture.

[43] Gao X., Jiang L., Xu J., Liu W., Li S., Huang W., Zhao H., Yang Z., Yu X., Wei Z. (2022). Aflatoxin B1-Activated Heterophil Extracellular Traps Result in the Immunotoxicity to Liver and Kidney in Chickens. Dev. Comp. Immunol..

[44] Liu N., Wang J.Q., Liu Z.Y., Wang Y.C., Wang J.P. (2018). Comparison of Probiotics and Clay Detoxifier on the Growth Performance and Enterotoxic Markers of Broilers Fed Diets Contaminated with Aflatoxin B1. J. Appl. Poult. Res..

[45] Mesgar A., Shahryar H.A., Bailey C.A., Ebrahimnezhad Y., Mohan A. (2022). Effect of Dietary L-Threonine and Toxin Binder on Performance, Blood Parameters, and Immune Response of Broilers Exposed to Aflatoxin B1. Toxins.

[46] Cao W., Yu P., Yang K.P., Cao D. (2022). Aflatoxin B1: Metabolism, Toxicology, and Its Involvement in Oxidative Stress and Cancer Development. Toxicol. Mech. Methods.

[47] Wang H., Li W., Muhammad I., Sun X., Cui X., Cheng P., Qayum A., Zhang X. (2018). Biochemical Basis for the Age-Related Sensitivity of Broilers to Aflatoxin B1. Toxicol. Mech. Methods.

[48] Li S., Muhammad I., Yu H., Sun X., Zhang X. (2019). Detection of Aflatoxin Adducts as Potential Markers and the Role of Curcumin in Alleviating AFB1-Induced Liver Damage in Chickens. Ecotoxicol. Environ. Saf..

[49] Wei J.T., Wu K.T., Sun H., Khalil M.M., Dai J.F., Liu Y., Liu Q., Zhang N.Y., Qi D.S., Sun L.H. (2019). A Novel Modified Hydrated Sodium Calcium Aluminosilicate (HSCAS) Adsorbent Can Effectively Reduce T-2 Toxin-Induced Toxicity in Growth Performance, Nutrient Digestibility, Serum Biochemistry, and Small Intestinal Morphology in Chicks. Toxins.

[50] Shah Alam M., Maowa Z., Das Subarna S., Hoque M.N. (2024). Mycotoxicosis and Oxidative Stress in Poultry: Pathogenesis and Therapeutic Insights. Worlds Poult. Sci. J..

[51] Abdel-Sattar W.M., Sadek K.M., Elbestawy A.R., Mourad D.M. (2019). The Protective Role of Date Palm (Phoenix Dactylifera Seeds) against Aflatoxicosis in Broiler Chickens Regarding Carcass Characterstics, Hepatic and Renal Biochemical Function Tests and Histopathology. J. Worlds Poult. Res..

[52] Sarker M.T., Wan X.L., Yang H.M., Wang Z.Y. (2023). AflatoxinB1 (AFB1) and Its Toxic Effect on the Broilers Intestine: A Review. Vet. Med. Sci..

[53] Poloni V., Magnoli A., Fochesato A., Cristofolini A., Caverzan M., Merkis C., Montenegro M., Cavaglieri L. (2020). A Saccharomyces Cerevisiae RC016-Based Feed Additive Reduces Liver Toxicity, Residual Aflatoxin B1 Levels and Positively Influences Intestinal Morphology in Broiler Chickens Fed Chronic Aflatoxin B1-Contaminated Diets. Anim. Nutr..

[54] Wang L., Huang Q., Wu J., Wu W., Jiang J., Yan H., Huang J., Sun Y., Deng Y. (2022). The Metabolism and Biotransformation of AFB1: Key Enzymes and Pathways. Biochem. Pharmacol..

[55] Neeff D.V., Ledoux D.R., Rottinghaus G.E., Bermudez A.J., Dakovic A., Murarolli R.A., Oliveira C.A.F. (2013). In Vitro and in Vivo Efficacy of a Hydrated Sodium Calcium Aluminosilicate to Bind and Reduce Aflatoxin Residues in Tissues of Broiler Chicks Fed Aflatoxin B1. Poult. Sci..

[56] Vila-Donat P., Marín S., Sanchis V., Ramos A.J. (2018). A Review of the Mycotoxin Adsorbing Agents, with an Emphasis on Their Multi-Binding Capacity, for Animal Feed Decontamination. Food Chem. Toxicol..

[57] Hernández-Martínez S.P., Delgado-Cedeño A., Ramos-Zayas Y., Franco-Molina M.A., Méndez-Zamora G., Marroquín-Cardona A.G., Kawas J.R. (2023). Aluminosilicates as a Double-Edged Sword: Adsorption of Aflatoxin B1 and Sequestration of Essential Trace Minerals in an In Vitro Gastrointestinal Poultry Model. Toxins.

[58] Rejeb R., de Baere S., Devreese M., Ducatelle R., Croubels S., Ayed M.H., Ghorbal A., Antonissen G. (2020). Calcination Improves the In Vivo Efficacy of a Montmorillonite Clay to Bind Aflatoxin G1 in Broiler Chickens: A Toxicokinetic Approach. Toxins.

[59] Bouhlali E.d.T., Hmidani A., Bourkhis B., Khouya T., Ramchoun M., Filali-Zegzouti Y., Alem C. (2020). Phenolic Profile and Anti-Inflammatory Activity of Four Moroccan Date (*Phoenix dactylifera* L.) Seed Varieties. Heliyon.

[60] Vázquez-Durán A., Nava-Ramírez M.d.J., Téllez-Isaías G., Méndez-Albores A. (2022). Removal of Aflatoxins Using Agro-Waste-Based Materials and Current Characterization Techniques Used for Biosorption Assessment. Front. Vet. Sci..

[61] Afifi H.S., Hashim I.B., Altubji S.I. (2017). Optimizing Extraction Conditions of Crude Fiber, Phenolic Compounds, Flavonoids and Antioxidant Activity of Date Seed Powder. J. Food Sci. Technol..

